# Bird–window collisions: A comprehensive dataset for the Neotropical region

**DOI:** 10.1002/ecy.70126

**Published:** 2025-06-13

**Authors:** Augusto João Piratelli, Bianca Costa Ribeiro, Wesley Dáttilo, Luis‐Bernardo Vázquez, Anelisa Ferreira de Almeida Magalhães, Edna Maria Gomes Cavalcante, Eric Silva, Giovanna Viana Cruz, Gisele Regina Ruy, Juliana Laurito Summa, Júlia Milan, Leila Pedrosa, Leticia Bolian Zimback, Marcello Nardi, Marcos Gonçalves da Silva, Pedro Rocha dos Santos, Sylvia Maria Matsuda, Diana Santa, Javier Garzón, Maria Angela Echeverry‐Galvis, Albert Ospina Duque, Oscar Humberto Marín Gómez, Martha Garro Cruz, Ignacio Gutiérrez, Luis Sandoval, Lucas Penna Soares Santos, Marcelo Ferreira de Vasconcelos, Bruno Petri, Fabio Dores, Haroldo Furuya, Lilian Sayuri Fitorra, Liliane Milanelo, Valéria Pedro, Rose Marie Menacho‐Odio, Natalia Ocampo‐Peñuela, Daniel Klem, Michelle García‐Arroyo, Miguel A. Gómez‐Martínez, Octavio Rojas‐Soto, Paulina Uribe‐Morfín, Johan Moreno‐Velasquez, Laura Agudelo‐Álvarez, Irma Ruan‐Tejeda, Sarahy Contreras‐Martínez, Vannia del Carmen Gomez‐Moreno, Camila Mazoni, Claudia Almeida Igayara Souza, Cristiane Espinosa Bolochio, David de Almeida Braga, Fernanda de Castro Magalhães, Gilberto Nogueira Penido‐Júnior, Hilari Wanderley Hidasi, Marcos Antônio Melo, Mariana Castanheira Grimaldi, Thais Caroline Sanches, Natalia Rebolo‐Ifrán, Santiago Niño‐Maldonado, David Ocampo, Orlando Acevedo‐Charry, Camilo E. Sánchez‐Sarria, Diego Cueva, Laura Ramírez Uribe, Sofía M. Alfonso‐Velasco, Ilse Esparza, Julian Avila‐Campos, Vitor Q. Piacentini, Flávia Chaves, Gabriele Andreia da Silva, Juliana Paulo da Silva, Michelle Baptista, Eduardo Roberto Alexandrino, Fabio de Mello Patiu, Yandry Hernandez, Leonardo Ordóñez‐Delgado, Jorge Valencia‐Herverth, Raúl Valencia‐Herverth, Camila Esser Tenfen, Thais Caroline Lopes de Oliveira, Nadezhda Bonilla‐S, Nicolas Tellez‐Colmenares, Iriana Zuria, Larissa D. Biasotto, Marcos Tokuda, Fernando González‐García, Juan Carlos Fernández‐Ordóñez, Thaís Brisque, Ivyson Aguiar, Victor Leandro‐Silva, Fábio M. Da Costa, Giovanna Marschner, Felipe A. Estela, Fabio Germán Cupul‐Magaña, Martha Gabriela Arroyo‐Joya, Augusto Batisteli, Rosane Costa, Rafael Calderón‐Parra, Patrícia Debrassi, Miguel Ángel Aguilar‐Gómez, Rubén Ortega‐Álvarez, Aura Puga‐Caballero, Lucila Castro, Juan F. Escobar‐Ibáñez, João Carlos Pena, Karlla Vanessa de Camargo Barbosa, Thiago Filadelfo, Ismael Franz, Alfredo Acosta‐Ramírez, Lucas Gonçalves da Silva, Alberto González‐Gallina, Alan Monroy‐Ojeda, Claudio Leite Novaes, Mariane C. Kaizer, Giuliano Müller Brusco, Crizanto Brito De‐Carvalho, Lucas Leveau, Santiago Santoandré, Carlos M. Leveau, Daniel Perrella, Ariadna Tobón‐Sampedro, Mateo López‐Victoria, Bruno Rodrigo de Albuquerque França, Alexander Vicente Christianini, Matilde Alfaro, Eliana Blanco Pérez, Ronald Armando Fernández‐Gómez, Breno Dias Vitorino, Marco Aurélio Pizo, Pamela Pairo, Allan Clé, Luz E. Zamudio‐Beltrán, George Mendes Taliaferro Mattox, Raone Mariano, Enzo Coletti‐Manzoli, Ian MacGregor‐Fors

**Affiliations:** ^1^ Departamento de Ciências Ambientais, CCTS Universidade Federal de São Carlos Sorocaba São Paulo Brazil; ^2^ Programa de Pós‐graduação em Biologia – Diversidade e manejo da vida silvestre Universidade do Vale do Rio dos Sinos São Leopoldo Rio Grande do Sul Brazil; ^3^ Red de Ecoetología, Instituto de Ecología, AC, INECOL Xalapa Veracruz Mexico; ^4^ Ecology, Landscape and Sustainability Group TAO, El Colegio de la Frontera Sur San Cristóbal de Las Casas Chiapas Mexico; ^5^ Divisão da Fauna Silvestre Secretaria do Verde e Meio Ambiente, Prefeitura da Cidade de São Paulo São Paulo São Paulo Brazil; ^6^ Fundación para la Investigación y Conservación de Especies Nativas Neotropicales Continentales y Oceánicas (FICENANCO) Salento Quindío Colombia; ^7^ Departamento de Ecología y Territorio, Facultad de Estudios Ambientales y Rurales Pontificia Universidad Javeriana Bogotá Cundinamarca Colombia; ^8^ CIBUQ, Centro de Estudios en Biodiversidad y Biotecnología del Quindío Universidad del Quindío Armenia Quindío Colombia; ^9^ Programa de Biología, Grupo de Investigación y Asesoría en Estadística Universidad del Quindío Armenia Quindío Colombia; ^10^ Universidad Estatal a Distancia Monteverde Puntarenas Costa Rica; ^11^ Laboratório de Ecología Urbana y Comunicación Animal, Escuela de Biología Universidad de Costa Rica Montes de Oca San José Costa Rica; ^12^ Instituto Chico Mendes de Conservação da Biodiversidade/Centro Nacional de Pesquisa e Conservação de Aves Silvestres (ICMBio/CEMAVE) Núcleo de Gestão Integrada de Fernando de Noronha Fernando de Noronha Pernambuco Brazil; ^13^ Museu de Ciências Naturais, Pontifícia Universidade Católica de Minas Gerais Belo Horizonte Minas Gerais Brazil; ^14^ Centro de Triagem de Animais Silvestres do Parque Ecológico do Tietê São Paulo São Paulo Brazil; ^15^ Programa de Manejo de Recursos Naturales, Escuela de Ciencias Exactas y Naturales Universidad Estatal a Distancia San José Costa Rica; ^16^ Environmental Studies Department University of California, Santa Cruz Santa Cruz California USA; ^17^ Acopian Center for Ornithology, Department of Biology Muhlenberg College Allentown Pennsylvania USA; ^18^ Faculty of Biological and Environmental Sciences University of Helsinki Lahti Finland; ^19^ Instituto de Biotecnología y Ecología Aplicada (INBIOTECA), Universidad Veracruzana Xalapa Veracruz Mexico; ^20^ Laboratorio de Bioclimatología, Red de Biología Evolutiva Instituto de Ecología, A. C. Xalapa Veracruz Mexico; ^21^ Escuela Nacional de Estudios Superiores ENES Unidad León Universidad Nacional Autónoma de México León Guanajuato Mexico; ^22^ Grupo de Observadores de Aves ANDIGENA, Pontificia Universidad Javeriana Bogotá Bogotá Colombia; ^23^ Departamento de Ciencias Naturales y Matemáticas Pontificia Universidad Javeriana Cali Cali Valle del Cauca Colombia; ^24^ Departamento de Ecología y Recursos Naturales‐IMECBIO, Centro Universitario de la Costa Sur Universidad de Guadalajara‐CUCostaSur Autlán de Navarro Jalisco Mexico; ^25^ División de Estudios de Posgrado Instituto Tecnologico de Ciudad Victoria Tamaulipas Victoria Tamaulipas Mexico; ^26^ Zoológico Municipal de Guarulhos Guarulhos São Paulo Brazil; ^27^ Programa de Pós‐Graduação em Ecologia e Recursos Naturais Universidade Federal de São Carlos São Carlos São Paulo Brazil; ^28^ Grupo de Investigaciones en Biología de la Conservación, Laboratorio Ecotono, INIBIOMA (Universidad Nacional del Comahue – CONICET) San Carlos de Bariloche Rio Negro Argentina; ^29^ Facultad de Ingeniería y Ciencias, Centro Universitario Victoria Universidad Autónoma de Tamaulipas Victoria Tamaulipas Mexico; ^30^ Department of Ecology and Evolutionary Biology Princeton University Princeton New Jersey USA; ^31^ Alliance Bioversity International and CIAT Palmira Valle del Cauca Colombia; ^32^ Instituto de Ciencias Naturales, Universidad Nacional de Colombia Bogotá Distrito Capital (D.C.) Colombia; ^33^ Department of Natural Resource Sciences McGill University Montreal Quebec Canada; ^34^ Departamento de Biologia e Zoologia & PPG Zoologia Instituto de Biociências Universidade Federal de Mato Grosso Cuiabá Mato Grosso Brazil; ^35^ Instituto Nacional da Mata Atlântica (INMA) Santa Teresa Espírito Santo Brazil; ^36^ Departamento de Ciências Florestais, Laboratório de Ecologia, Manejo e Conservação da Fauna Silvestre Universidade de São Paulo, Escola Superior de Agricultura “Luiz de Queiroz” Piracicaba São Paulo Brazil; ^37^ Self‐Employed Biologist Rio de Janeiro Rio de Janeiro Brazil; ^38^ Escuela de Ciencias Exactas y Naturales Universidad Estatal a Distancia Peréz Zeledón San Jose Costa Rica; ^39^ Laboratorio de Ecología Tropical y Servicios Ecosistémicos ‐ EcoSs Lab, Departamento de Ciencias Biológicas y Agropecuarias Universidad Técnica Particular de Loja Loja Ecuador; ^40^ Tecnológico Nacional de México, Instituto Tecnológico de Huejutla Huejutla de Reyes Hidalgo Mexico; ^41^ Universidade Tecnológica Federal do Paraná Dois Vizinhos Paraná Brazil; ^42^ Instituto de Ciencias Naturales (ICN), Grupo de Ornitología de la Universidad Nacional de Colombia Bogotá Cundinamarca Colombia; ^43^ Laboratorio de Interacciones, Centro de Investigaciones Biológicas Universidad Autónoma del Estado de Hidalgo Pachuca Hidalgo Mexico; ^44^ BirdLife International, The David Attenborough Building Cambridge UK; ^45^ Parque Zoológico Municipal Quinzinho de Barros Sorocaba São Paulo Brazil; ^46^ Red Biología y Conservación de Vertebrados, Instituto de Ecología, AC, INECOL Xalapa Veracruz Mexico; ^47^ Fundación Científica ARA MACAO San Carlos Cojedes Venezuela; ^48^ Instituto do Meio Ambiente de Santa Catarina Florianópolis Santa Catarina Brazil; ^49^ Parque Estadual Dois Irmãos Recife Pernambuco Brazil; ^50^ Laboratório de Ecologia e Evolução de aves, Centro de Biociências Universidade Federal de Pernambuco Recife Pernambuco Brazil; ^51^ Museu de Ciências Naturais da Universidade de Caxias do Sul, Universidade de Caxias do Sul Caxias do Sul Rio Grande do Sul Brazil; ^52^ Centro de Ciências da Vida Universidade de Caxias do Sul Caxias do Sul Rio Grande do Sul Brazil; ^53^ Centro Universitario de la Costa Universidad de Guadalajara (UdeG) Puerto Vallarta Jalisco Mexico; ^54^ Pós‐doutoramento, Departamento de Hidrobiologia Universidade Federal de São Carlos São Carlos São Paulo Brazil; ^55^ Consultant Mexico City Mexico Mexico; ^56^ Special Education Teacher Balneário Camboriú Santa Catarina Brazil; ^57^ Comisión Nacional para el Conocimiento y Uso de la Biodiversidad Mexico City Mexico Mexico; ^58^ Investigadoras e Investigadores por México del Consejo Nacional de Ciencia y Tecnología (CONACYT), Dirección Regional Occidente Guadalajara Jalisco Mexico; ^59^ Laboratorio de Ecología, Unidad de Biología, Tecnología y Prototipos, Facultad de Estudios Superiores Iztacala‐UNAM Los Reyes Iztacala Estado de México México; ^60^ Sítio Encanto de Roça Itabirito Minas Gerais Brazil; ^61^ Gnósis ‐ Naturaleza con Ciencia A.C. Guadalajara Jalisco Mexico; ^62^ Doctorado en Ciencias de la Sustentabilidad Universidad Rosario Castellanos de la Ciudad de México Ciudad de México Mexico; ^63^ Araucaria Sustenability and Innovation Lab (LASI) Environmental Studies Center (CEA), São Paulo State University (UNESP) Rio Claro São Paulo Brazil; ^64^ Qualis Consultoria Ambiental Grupo de Pesquisa e Conservação da Arara‐azul‐de‐lear Lauro de Freitas Bahia Brazil; ^65^ Departamento de Zoologia Instituto de Biociências, Universidade Federal do Rio Grande do Sul Porto Alegre Rio Grande do Sul Brazil; ^66^ Centro Universitario Universidad Autónoma de Querétaro Santiago de Querétaro Querétaro Mexico; ^67^ Campus UnB, Asa Norte, Universidade de Brasília Brasília Distrito Federal Brazil; ^68^ Kiekari Bird Observatory, Kiekari Terra A. C. Xico Veracruz Mexico; ^69^ Rede Eco‐Diversa para Conservação da Biodiversidade Tombos Minas Gerais Brazil; ^70^ School of Science, Engineering, and Environment University of Salford‐Manchester Salford UK; ^71^ Laboratório de Aves Aquáticas e Tartarugas Marinhas Universidade Federal do Rio Grande Rio Grande Rio Grande do Sul Brazil; ^72^ Laboratório de Aves Aquáticas e Tartarugas Marinhas Universidade Federal do Rio Grande Porto Alegre Rio Grande do Sul Brazil; ^73^ Instituto Canindé de Pesquisa e Conservação Gama Distrito Federal Brazil; ^74^ Departamento de Ecología, Genética y Evolución, Facultad de Ciencias Exactas y Naturales Universidad de Buenos Aires—IEGEBA (CONICET‐UBA) Buenos Aires Argentina; ^75^ Instituto de Producción, Economía y Trabajo Universidad Nacional de Lanús Lanús Argentina; ^76^ Consejo Nacional de Investigaciones Científicas y Técnicas Buenos Aires Argentina; ^77^ Consultant São Paulo São Paulo Brazil; ^78^ Jardín Etnobotánico Francisco Peláez R San Andrés Cholula Puebla Mexico; ^79^ Facultad de Ingeniería y Ciencias Pontificia Universidad Javeriana Cali Cali Valle del Cauca Colombia; ^80^ PLANOAMBIENTAL – Planejamento e Estudos Ambientais Ltda Natal Rio Grande do Norte Brazil; ^81^ Departamento de Ecología y Gestión Ambiental Centro Universitario Regional del Este, Universidad de la República Maldonado Maldonado Uruguay; ^82^ Laboratorio de Biología de Organismos Centro de Ecología, Instituto Venezolano de Investigaciones Científicas Altos de Pipe Miranda Venezuela; ^83^ Instituto de Neuroetología, Universidad Veracruzana Xalapa Veracruz Mexico; ^84^ Grupo de Investigación en Ecología Evolutiva, Departamento de Biología Universidad de Nariño Pasto Nariño Colombia; ^85^ Centro de Pesquisa em Limnologia, Biodiversidade e Etnobiologia do Pantanal Universidade do Estado de Mato Grosso Cáceres Mato Grosso Brazil; ^86^ Centro de Pesquisa em Biodiversidade e Mudanças Climáticas (CBioClima), Departamento de Biodiversidade, Instituto de Biociências Universidade Estadual Paulista Rio Claro São Paulo Brazil; ^87^ Programa de Pós‐graduação em Aquicultura e Pesca no Instituto de Pesca de São Paulo (IP) São Paulo São Paulo Brazil; ^88^ Departamento de Biología Evolutiva, Facultad de Ciencias, Museo de Zoología Universidad Nacional Autónoma de México Mexico City Mexico Mexico; ^89^ Laboratório de Ictiologia de Sorocaba, CCHB Universidade Federal de São Carlos Sorocaba São Paulo Brazil; ^90^ Universidade Federal de São Carlos Sorocaba São Paulo Brazil

**Keywords:** avifauna, biodiversity, bird conservation, bird strikes, human‐made structures, Neotropical birds, urban ecology, urbanization, window panes

## Abstract

Our primary objective was to compile a comprehensive dataset on bird–window collisions throughout the Neotropical region, including both published and unpublished sources. On May 12, 2020, we extensively disseminated invitations to provide data via email and social media platforms. By providing a template worksheet, we required standardized information from collaborators to complete and register their data. To better understand how these data were acquired (e.g., incidental observations and systematic procedures), we sent out a survey to all collaborators. We established rigorous validation criteria for data inclusion and conducted thorough curation procedures to ensure accuracy. After the filtering process, we compiled a total of 4103 bird–window collision reports. These came from 11 Neotropical countries, dating from 1946 to 2020, and revealing distinct regional patterns and potential seasonal patterns. The five most frequent orders were Passeriformes (2451), Columbiformes (520), Apodiformes (377), Psittaciformes (202), and Piciformes (186). Data on bird–window collisions were collected through a local specific systematic protocol (1419), by chance (1252), by government agencies (742), and by other approaches (632), while a few reports were collected by unknown procedures (58). The volume of records across months in our dataset suggests that there may be temporal patterns, with peaks: the first one in March–April and the second one in October–November, which seem to align with the major migration and reproduction seasons. This dataset represents the first comprehensive effort in the Neotropical region focused on bird–window collision data, providing valuable insights for further scientific advancements and conservation policies. The data are free from copyright or proprietary restrictions. Please cite this data paper when using the data in publications or scientific presentations.

## CONFLICT OF INTEREST STATEMENT

The authors declare no conflicts of interest.

## Supporting information


Data S1.

## Data Availability

The dataset is available as Supporting Information (Data S1) and is also available in Zenodo at https://doi.org/10.5281/zenodo.15271126.

