# Supplementary material for: Bird–window collisions: A comprehensive dataset for the Neotropical region
Source: Ecology. 2025 Jun 13;106(6):e70126. doi: 10.1002/ecy.70126 (PMC12163362; doi:10.1002/ecy.70126)

Appendix S1

Bird–window collisions: A comprehensive dataset for the Neotropical region

Augusto João Piratelli, Bianca Costa Ribeiro, Wesley Dáttilo, Luis-Bernardo Vázquez, Anelisa Ferreira de Almeida Magalhães, Edna Maria Gomes Cavalcante, Eric Silva, Giovanna Viana Cruz, Gisele Regina Ruy, Juliana Laurito Summa, Júlia Milan, Leila Pedrosa, Leticia Bolian Zimback, Marcello Nardi, Marcos Gonçalves da Silva, Pedro Rocha dos Santos, Sylvia Maria Matsuda, Diana Santa, Javier Garzón, Maria Angela Echeverry-Galvis, Albert Ospina Duque, Oscar Humberto Marín Gómez, Martha Garro Cruz, Ignacio Gutiérrez, Luis Sandoval, Lucas Penna Soares Santos, Marcelo Ferreira de Vasconcelos, Bruno Petri, Fabio Dores, Haroldo Furuya, Lilian Sayuri Fitorra, Liliane Milanelo, Valéria Pedro, Rose Marie Menacho-Odio, Natalia Ocampo-Peñuela, Daniel Klem Jr., Michelle García-Arroyo, Miguel A. Gómez-Martínez, Octavio Rojas-Soto, Paulina Uribe-Morfín, Johan Moreno-Velasquez, Laura Agudelo-Álvarez, Irma Ruan-Tejeda, Sarahy Contreras-Martínez, Vannia del Carmen Gomez-Moreno, Camila Mazoni, Claudia Almeida Igayara Souza, Cristiane Espinosa Bolochio, David de Almeida Braga, Fernanda de Castro Magalhães, Gilberto Nogueira Penido-Júnior, Hilari Wanderley Hidasi, Marcos Antônio Melo, Mariana Castanheira Grimaldi, Thais Caroline Sanches, Natalia Rebolo-Ifrán, Santiago Niño-Maldonado, David Ocampo, Orlando Acevedo-Charry, Camilo E. Sánchez-Sarria, Diego Cueva, Laura Ramírez Uribe, Sofía M. Alfonso-Velasco, Ilse Esparza, Julian Avila-Campos, Vitor Q. Piacentini, Flávia Chaves, Gabriele Andreia da Silva, Juliana Paulo da Silva, Michelle Baptista, Eduardo Roberto Alexandrino, Fabio de Mello Patiu, Yandry Hernandez, Leonardo Ordóñez-Delgado, Jorge Valencia-Herverth, Raúl Valencia-Herverth, Camila Esser Tenfen, Thais Caroline Lopes de Oliveira, Nadezhda Bonilla-S, Nicolas Tellez-Colmenares, Iriana Zuria, Larissa D. Biasotto, Marcos Tokuda, Fernando González-García, Juan Carlos Fernández-Ordóñez, Thaís Brisque, Ivyson Aguiar, Victor Leandro-Silva, Fábio M. Da Costa, Giovanna Marschner, Felipe A. Estela, Fabio Germán Cupul-Magaña, Martha Gabriela Arroyo-Joya, Augusto Batisteli, Rosane Costa, Rafael Calderón-Parra, Patrícia Debrassi, Miguel Ángel Aguilar-Gómez, Rubén Ortega-Álvarez, Aura Puga-Caballero, Lucila Castro, Juan F. Escobar-Ibáñez, João Carlos Pena, Karlla Vanessa de Camargo Barbosa, Thiago Filadelfo, Ismael Franz, Alfredo Acosta-Ramírez, Lucas Gonçalves da Silva, Alberto González-Gallina, Alan Monroy-Ojeda, Claudio Leite Novaes, Mariane C. Kaizer, Giuliano Müller Brusco, Crizanto Brito De-Carvalho, Lucas Leveau, Santiago Santoandré, Carlos M. Leveau, Daniel Perrella, Ariadna Tobón-Sampedro, Mateo López-Victoria, Bruno Rodrigo de Albuquerque França, Alexander Vicente Christianini, Matilde Alfaro, Eliana Blanco Pérez, Ronald Armando Fernández-Gómez, Breno Dias Vitorino, Marco Aurélio Pizo, Pamela Pairo, Allan Clé, Luz E. Zamudio-Beltrán, George Mendes Taliaferro Mattox, Raone Mariano, Enzo Coletti-Manzoli, Ian MacGregor-Fors

Fig. S1. Examples of how data collection was communicated via email and social media.

a) Initial email (recipient addresses omitted for privacy reasons).

DATA PAPER: BIRD-WINDOW
COLLISIONS IN LATIN AMERICA

LECO

Dear collaborators,

This is the first update on the data paper "Bird-Window Collisions in Latin America". Our mission is to join the largest data set on these accidents throughout Latin America. We would like to count on your contribution to this data paper. **We will accept reliable records that have at least species-level identification, date (year, month, day), and geographic information (geographic coordinates, site, city, state/province, country).** Please, look for further information at the “explanations” tab in the attached spreadsheet to check how to properly enter your data in the table. Do not change the header of the spreadsheet. Enter as many records and locations as you have, and if no information is available, fill empty cells with NA. The geographic coordinate must by as much precise as possible. The validation of this data set will allow the inclusion of the collaborator as a co-author in this paper, which will be submitted to “Ecology”.

The deadline for sending you datasheet is July 12th, 2020.

Augusto Piratelli and Bianca Ribeiro are going to coordinate this project. If you have any further questions, please do not hesitate to contact us.
The preferred contact is through the e-mail: projectbirdwindowcollisions@gmail.com
Please forward this email to your collaborators.
Thank you very much in advance.

Prof. Dr. Augusto J. Piratelli
Bianca C. Ribeiro

1Depto. Ciências Ambientais, CCTS, Universidade Federal de São Carlos, Sorocaba, Brazil.
2Laboratório de Ecologia e Conservação, LECO, Univ. Federal de São Carlos, Sorocaba, Brazil.

b) First electronic flyer (in English).

DATA PAPER ECOLOGY
First update on the data paper "Bird-window collisions in Latin America"

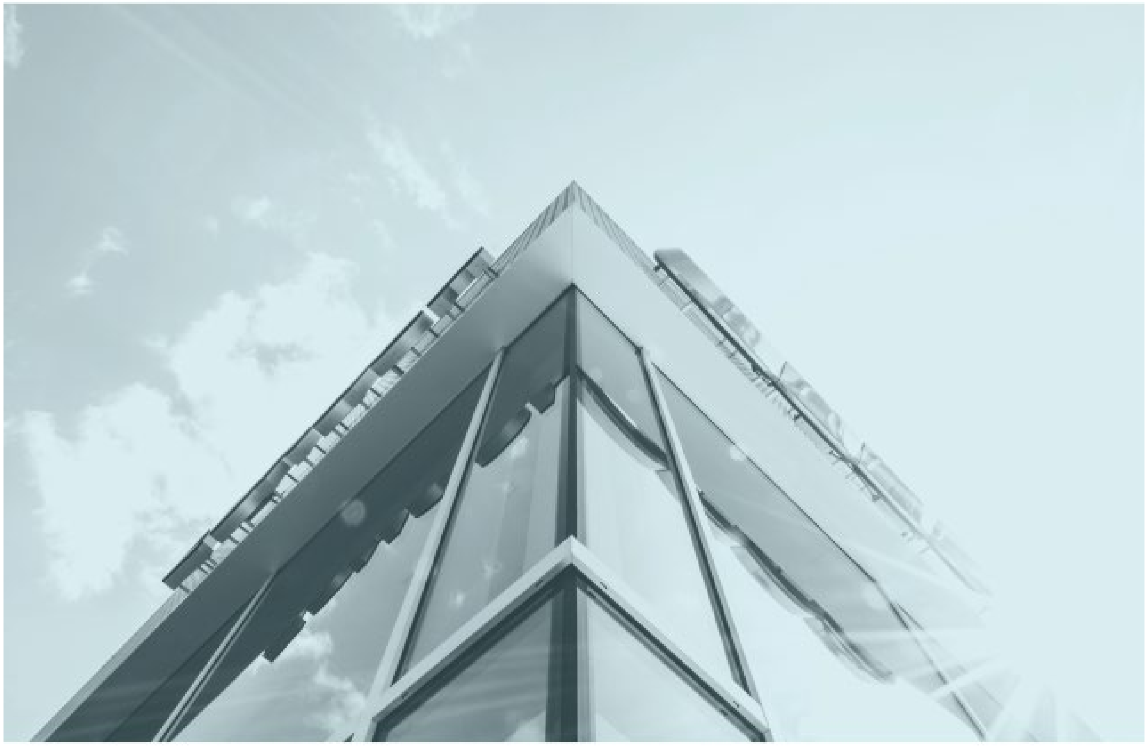

BIRD-WINDOW COLLISIONS IN LATIN AMERICA

Data paper for Ecology

Augusto J. Piratelli and Bianca C. Ribeiro
Federal University of São Carlos, São Paulo, Brazil

b) First electronic flyer (in English, continued).

FIRST UPDATE

This is the first update on the data paper "Bird-Window Collisions in Latin America". Our mission is to join the largest data set on these accidents throughout Latin America. We would like to count on your contribution to this data paper.

We will accept reliable records that have at least **species-level identification, date (year, month, day), and geographic information (geographic coordinates, site, city, state/province, country)**.

Attached to this email is an Excel spreadsheet to be filled in with the data. For you to be attached as a co-author of the publication, **you must fill in the minimum data described above**.

Deadline for sending you datasheet is July 12th, 2020.

This data paper is an initiative of researchers from the Ecology and Conservation Lab of UFSCar Sorocaba (LECO).

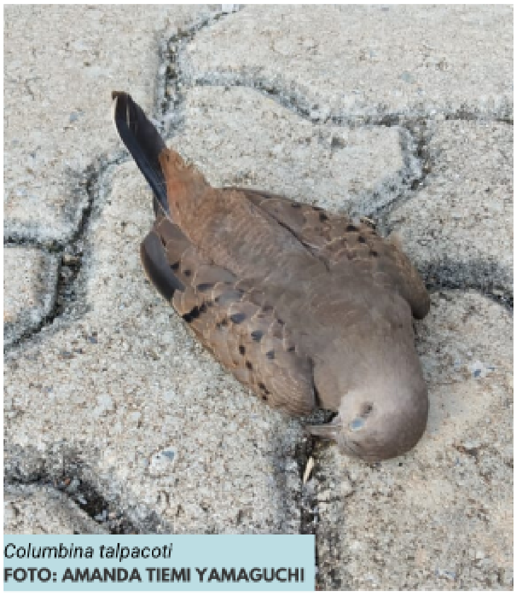

Columbina talpacoti
FOTO: AMANDA TIEMI YAMAGUCHI

WHO CAN BE A CO-AUTHOR?

Our goal is to collect reliable data on bird-window collisions across Latin America. Researchers, universities, institutions, and citizen scientists. Anyone who has the minimum data required can contact us.

CONTACT US

projectbirdwindowcollisions@gmail.com

@lecoaves

/lecoufscar

c) Second electronic flyer (in Spanish and Portuguese).

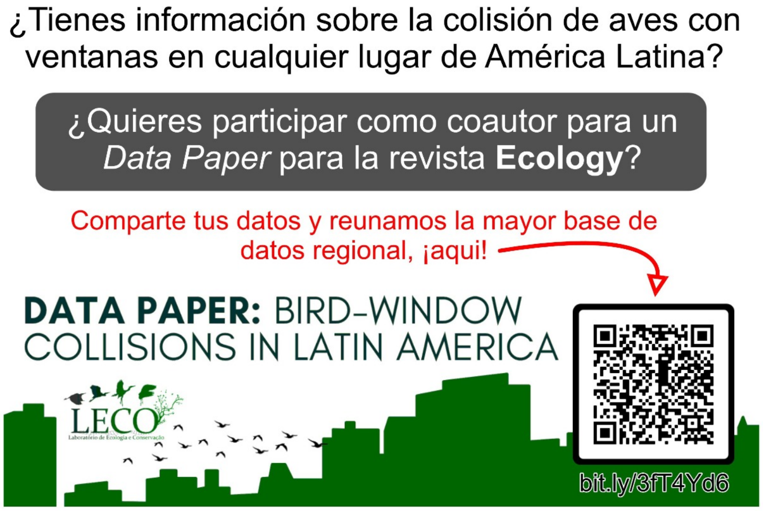

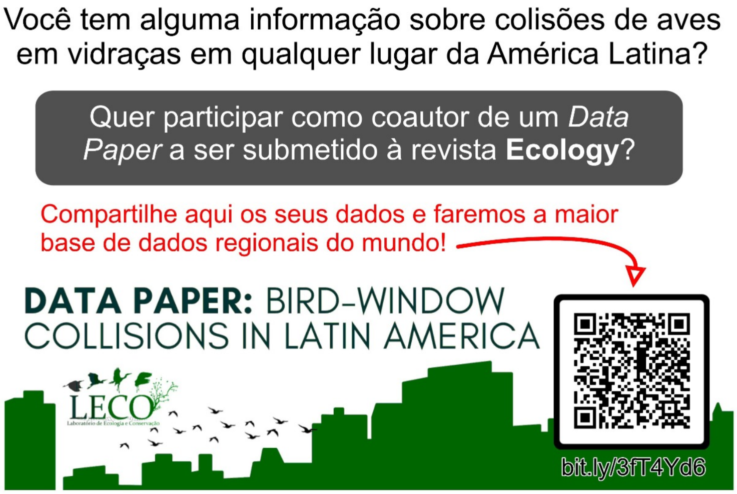

d) Third electronic flyer (in English).

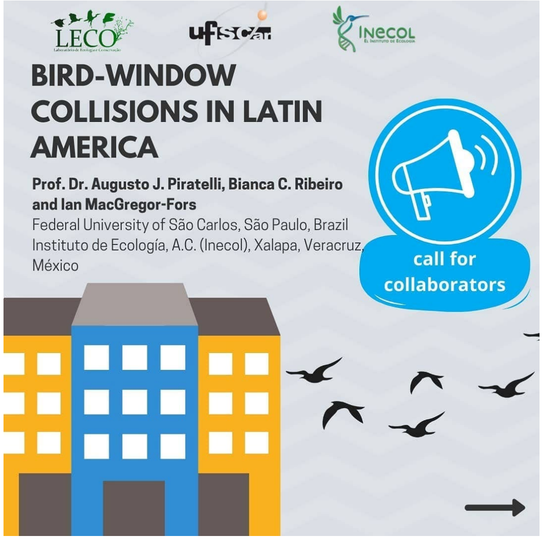

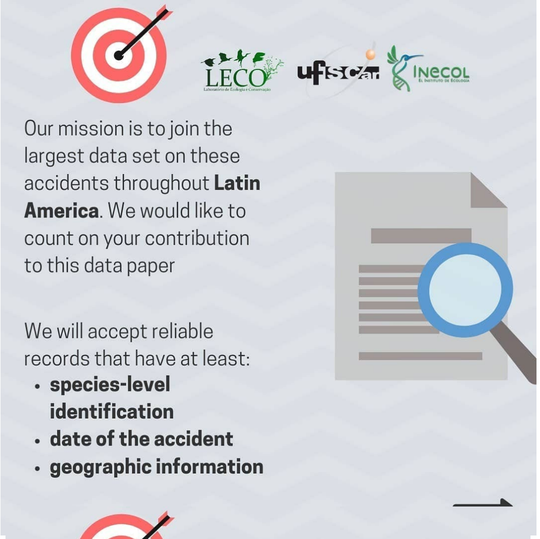

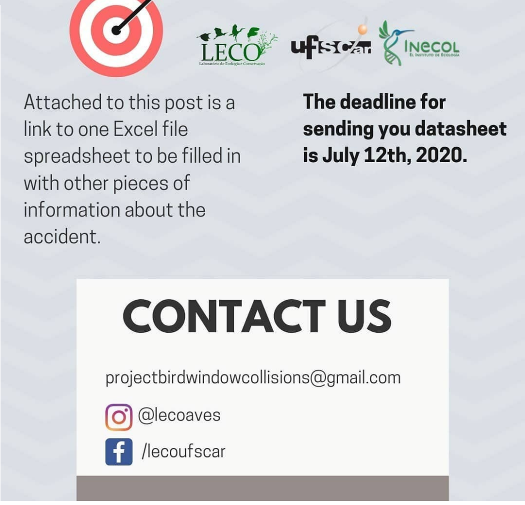

Supplement: Supplementary file 1 — Data S1. [file ECY-106-e70126-s001.zip › Appendix_S1.pdf]
