# Supplementary material for: Bird–window collisions: A comprehensive dataset for the Neotropical region
Source: Ecology. 2025 Jun 13;106(6):e70126. doi: 10.1002/ecy.70126 (PMC12163362; doi:10.1002/ecy.70126)
Supplement: Supplementary file 1 — Data S1. [file ECY-106-e70126-s001.zip › Metadata_S1.pdf]

Data Paper published in *Ecology*, an Ecological Society of America journal

Metadata S1

Bird–window collisions: A comprehensive dataset for the Neotropical region

Augusto João Piratelli, Bianca Costa Ribeiro, Wesley Dáttilo, Luis-Bernardo Vázquez, Anelisa Ferreira de Almeida Magalhães, Edna Maria Gomes Cavalcante, Eric Silva, Giovanna Viana Cruz, Gisele Regina Ruy, Juliana Laurito Summa, Júlia Milan, Leila Pedrosa, Leticia Bolian Zimback, Marcello Nardi, Marcos Gonçalves da Silva, Pedro Rocha dos Santos, Sylvia Maria Matsuda, Diana Santa, Javier Garzón, Maria Angela Echeverry-Galvis, Albert Ospina Duque, Oscar Humberto Marín Gómez, Martha Garro Cruz, Ignacio Gutiérrez, Luis Sandoval, Lucas Penna Soares Santos, Marcelo Ferreira de Vasconcelos, Bruno Petri, Fabio Dores, Haroldo Furuya, Lilian Sayuri Fitorra, Liliane Milanelo, Valéria Pedro, Rose Marie Menacho-Odio, Natalia Ocampo-Peñuela, Daniel Klem Jr., Michelle García-Arroyo, Miguel A. Gómez-Martínez, Octavio Rojas-Soto, Paulina Uribe-Morfín, Johan Moreno-Velasquez, Laura Agudelo-Álvarez, Irma Ruan-Tejeda, Sarahy Contreras-Martínez, Vannia del Carmen Gomez-Moreno, Camila Mazoni, Claudia Almeida Igayara Souza, Cristiane Espinosa Bolochio, David de Almeida Braga, Fernanda de Castro Magalhães, Gilberto Nogueira Penido-Júnior, Hilari Wanderley Hidasi, Marcos Antônio Melo, Mariana Castanheira Grimaldi, Thais Caroline Sanches, Natalia Rebolo-Ifrán, Santiago Niño-Maldonado, David Ocampo, Orlando Acevedo-Charry, Camilo E. Sánchez-Sarria, Diego Cueva, Laura Ramírez Uribe, Sofía M. Alfonso-Velasco, Ilse Esparza, Julian Avila-Campos, Vitor Q. Piacentini, Flávia Chaves, Gabriele Andreia da Silva, Juliana Paulo da Silva, Michelle Baptista, Eduardo Roberto Alexandrino, Fabio de Mello Patiu, Yandry Hernandez, Leonardo Ordóñez-Delgado, Jorge Valencia-Herverth, Raúl Valencia-Herverth, Camila Esser Tenfen, Thais Caroline Lopes de Oliveira, Nadezhda Bonilla-S, Nicolas Tellez-Colmenares, Iriana Zuria, Larissa D. Biasotto, Marcos Tokuda, Fernando González-García, Juan Carlos Fernández-Ordóñez, Thaís Brisque, Ivyson Aguiar, Victor Leandro-Silva, Fábio M. Da Costa, Giovanna Marschner, Felipe A. Estela, Fabio Germán Cupul-Magaña, Martha Gabriela Arroyo-Joya, Augusto Batisteli, Rosane Costa, Rafael Calderón-Parra, Patrícia Debrassi, Miguel Ángel Aguilar-Gómez, Rubén Ortega-Álvarez, Aura Puga-Caballero, Lucila Castro, Juan F. Escobar-Ibáñez, João Carlos Pena, Karlla Vanessa de Camargo Barbosa, Thiago Filadelfo, Ismael Franz, Alfredo Acosta-Ramírez, Lucas Gonçalves da Silva, Alberto González-Gallina, Alan Monroy-Ojeda, Claudio Leite Novaes, Mariane C. Kaizer, Giuliano Müller Brusco, Crizanto Brito De-Carvalho, Lucas Leveau, Santiago Santoandré, Carlos M. Leveau, Daniel Perrella, Ariadna Tobón-Sampedro, Mateo López-Victoria, Bruno Rodrigo de Albuquerque França, Alexander Vicente Christianini, Matilde Alfaro, Eliana Blanco Pérez, Ronald Armando Fernández-Gómez, Breno Dias Vitorino, Marco Aurélio Pizo, Pamela Pairo, Allan Clé, Luz E. Zamudio-Beltrán, George Mendes Taliaferro Mattox, Raone Mariano, Enzo Coletti-Manzoli, Ian MacGregor-Fors

INTRODUCTION

The fragmentation and conversion of natural landscapes into agricultural and urban environments have given rise to numerous adverse impacts on biodiversity, frequently jeopardizing the populations of the most sensitive species (Gaston et al., 2003; Maxwell et al., 2016). Urbanization, characterized as a transformative land-use change process, alongside the complex metabolism of cities, has been recognized as a primary driver of global environmental changes (Sol et al., 2014; Palacio et al., 2018; Uribe-Morfín et al., 2021). Species filtering exhibits a non-random pattern; the decline in both phylogenetic and functional diversity observed within urban ecosystems, compared to the broader regional species pool, drives shift towards the prevalence of habitat- and diet generalist species (La Sorte et al., 2018; MacGregor-Fors et al., 2022).

Birds inhabiting urban systems face a plethora of threats, including shifts in parasite-host dynamics involving novel parasites and diseases (Jiménez-Peñuela et al., 2021) and attacks from non-native predators (e.g., cats and dogs) (Rebolo-Ifrán et al., 2021). When combined with habitat reduction, these factors can lead to decreasing abundance and even extinction of local populations. Moreover, human-made structures such as buildings, roads, and power transmission lines have also been shown to be significant causes of bird mortality (Santiago-Alarcon & Delgado-V, 2017). Modern urban landscapes have integrated high-rise buildings into their skylines, often using large panels of translucent or reflective glass (Al-Kodmany, 2013) that create one of the primary sources of anthropogenic avian fatalities (Klem, 2006, 2015). Estimates for Canada and the USA alone report more than one billion bird deaths per year (Loss et al., 2014; Sabo et al., 2016). Thus, given that it is nearly impossible to eradicate glass as a prevalent material in urban landscaping, deterrent management needs to be tested and implemented to promote bird-friendly cities (Rössler et al., 2015). Better strategies to address bird–window collisions could be a key factor in sustaining urban avifauna, its ecological functions, and human welfare.

While the impact of bird–window collisions has been highlighted for more than a decade in the USA and Canada; little – yet increasing – attention has been given to this crisis worldwide (but see Rösller et al., 2015; Mitrus & Zbyryt, 2018; Żmihorski et al., 2022). Recently, the Neotropical region has begun making progress in uncovering the impact of these structures on birds (e.g., Uribe-Morfín et al., 2021; Lee et al., 2024). Since 2010, some local studies have been developed, shedding light on this alarming threat in both urban and non-urban sites, and testing some preventive devices (e.g., Agudelo et al., 2010; Gómez-Martínez et al., 2019; Ribeiro and Piratelli, 2020). The properties that may either catalyze or mitigate these accidents remain unclear, and discussions aiming at the development of public policies to reduce bird–window collisions are rare (Basilio et al., 2020).

The Neotropical region is home to the highest avian diversity in the world, even within the urban context (MacGregor-Fors & Escobár Ibáñez, 2017), which increases the adverse impacts of the knowledge gap concerning bird–window collisions. This data paper compiles data from

over 100 collaborators across 11 countries within the Neotropical region. Data were voluntarily provided by collaborators after wide dissemination of the intention to publish this data paper (Appendix S1: Fig. S1), via social media and email. The data were collected mainly according to local specific systematic protocols, but also include anecdotal reports.

Class I. Dataset descriptors

A. ***Dataset identity***: Bird–window collisions: A comprehensive dataset for the Neotropical region

B. ***Dataset identification code***: BWC_Neotropical_region.csv

C. ***Dataset description***:
   1. **Principal investigators**: Augusto João Piratelli, Bianca Costa Ribeiro, Ian MacGregor-Fors
   2. **Abstract**: Our primary objective was to compile a comprehensive dataset on bird–window collisions throughout the Neotropical region, including both published and unpublished sources. On May 12, 2020, we extensively disseminated invitations to provide data via email and social media platforms. By providing a template worksheet, we required standardized information from collaborators to complete and register their data. To better understand how these data were acquired (e.g., incidental observations, systematic procedures), we sent out a survey to all collaborators. We established rigorous validation criteria for data inclusion and conducted thorough curation procedures to ensure accuracy. After the filtering process, we compiled a total of 4103 bird–window collision reports. These came from 11 Neotropical countries, dating from 1946–2020, and revealing distinct regional patterns and potential seasonal patterns. The five most frequent orders were Passeriformes (2451), Columbiformes (520), Apodiformes (377), Psittaciformes (202), and Piciformes (186). Data on bird–window collisions were collected through a local specific systematic protocol (1419), by chance (1252), by government agencies (742), and by other approaches (632), while a few reports were collected by unknown procedures (58). The volume of records across months in our dataset suggests that there may be temporal patterns, with peaks: the first one in March-April and the second one in October-November, which seem to align with the major migration and reproduction seasons. This dataset represents the first comprehensive effort in the Neotropical region focused on bird–window collision data, providing valuable insights for further scientific advancements, and conservation policies.
   3. **Keywords**: Avifauna; biodiversity; bird strikes; bird conservation; human-made structures; Neotropical birds; urbanization; urban ecology; window panes.

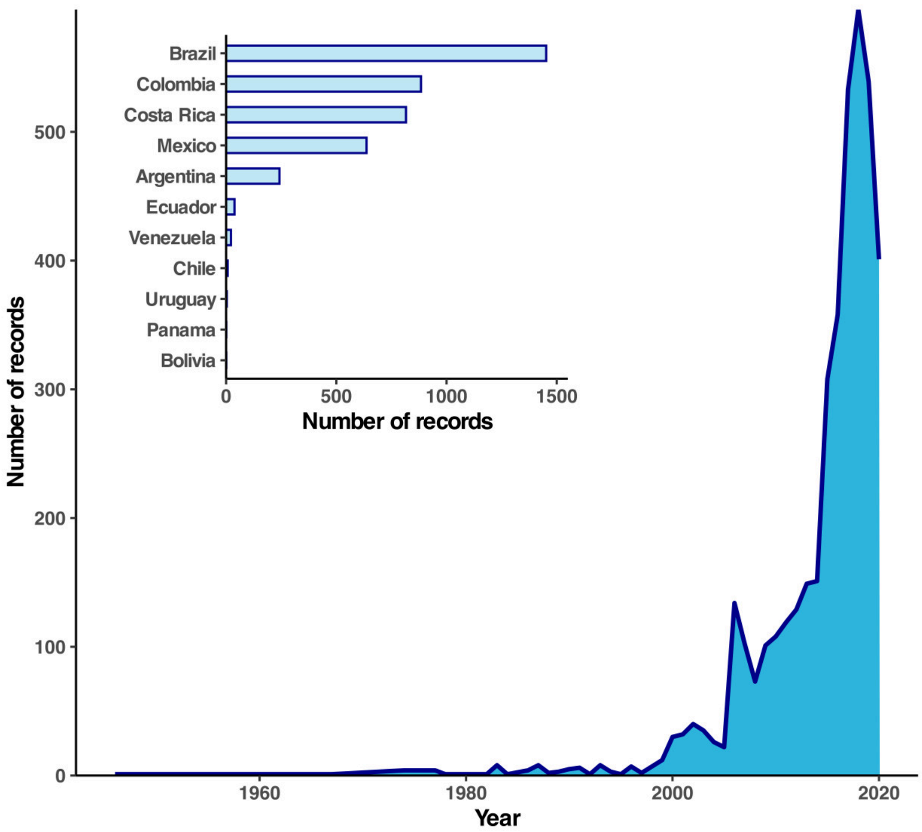

Figure 1. Number of bird–window collision records across the Neotropical region (1946–2020).

4. **Description**: Our main goal was to compile a comprehensive dataset on bird–window collisions throughout the Neotropical region, both published and unpublished. For this, we extensively disseminated multilingual invitations to provide data via email and across social media platforms (i.e., Spanish, Portuguese, and English) starting on May 12, 2020 (Appendix S1: Fig. S1). The sent messages included a QR code and website link with the instructions to share the data, including author contributions and a template for sharing bird-collision records and metadata for each cell. We established rigorous validation criteria for data inclusion and conducted thorough curation procedures to ensure accuracy. The curation procedure was performed mainly by AJP, BCR, and IM-F who additionally contacted regional ornithological experts. After the filtering process, we compiled a total of 4103 bird–window collision reports spanning from September 1946 to July 2020. To better understand how these data were acquired (e.g., by chance, systematically), we sent out a survey to all collaborators after initial data collection to clarify how the data were collected. An important proportion of the reported bird–window collisions were collected through a local

specific systematic protocol (1419), while 1252 reports were collected by chance, 742 by government agencies, 632 were collected through other approaches, and 58 reports were collected by unknown procedures. The data contained in the dataset includes eleven countries in the Neotropical region (Fig. 1 & 2), listed in descending order of representation: Brazil (1452 records), Colombia (884 records), Costa Rica (816 records), Mexico (637 records), Argentina (242 records), Ecuador (38 records), Venezuela (22 records), Chile (7 records), Uruguay (3 records), Panama (1 records), Bolivia (1 records). Among the 267 localities for which data were provided, São Paulo (Brazil, 629 records), Monteverde (Costa Rica, 548 records), and Bogotá (Colombia, 392 records) accounted for the highest number of bird–window strikes (Fig. 3).

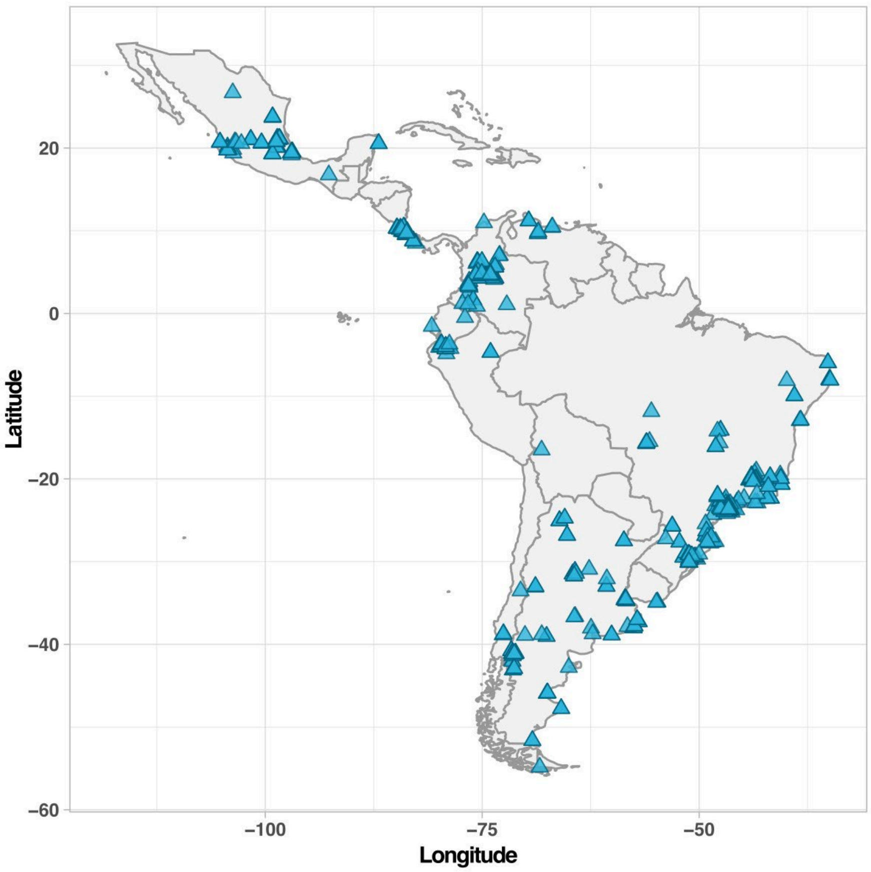

Figure 2. Geographic distribution of the 4,103 bird–window collision records across the Neotropical region (1946–2020).

There were records throughout the year, with apparent peaks in March-April and October-November (Fig. 4). However, we emphasize that the period of collision records spans from September 1946 to July 2020, with studies conducted at

different times of the year, which may limit temporal interpretation. While some studies are standardized, the time periods during which the collisions occurred are not, so temporal patterns should be interpreted with caution. We recognize temporal and local variations as potential sources of uncontrolled variability, which may represent a limitation of our database. Taxonomically, the five orders appearing most frequent in the dataset were: Passeriformes (2451 records), Columbiformes (520 records), Apodiformes (377 records), Psittaciformes (202 records), and Piciformes (186 records). In these orders, collisions mainly impacted birds from families Turdidae (Passeriformes; 757 records), Columbidae (Columbiformes; 520 records), Thraupidae (Passeriformes, 427 records), and Trochilidae (Apodiformes, 364 records). Across the collected data, there are four species with over 100 records: Swainson's Thrush (*Catharus ustulatus*, Turdidae, Passeriformes; 213 records)*,* Ruddy Ground Dove (*Columbina talpacoti*, Columbidae, Columbiformes; 134 records), Rufous-bellied Thrush (*Turdus rufiventris*, Turdidae, Passeriformes; 125 records), and Red-eyed Vireo (*Vireo olivaceus*, Vireonidae, Passeriformes; 118 records) are largely responsible for the bias in the greater number of collisions in their respective families and genera (Fig. 5).

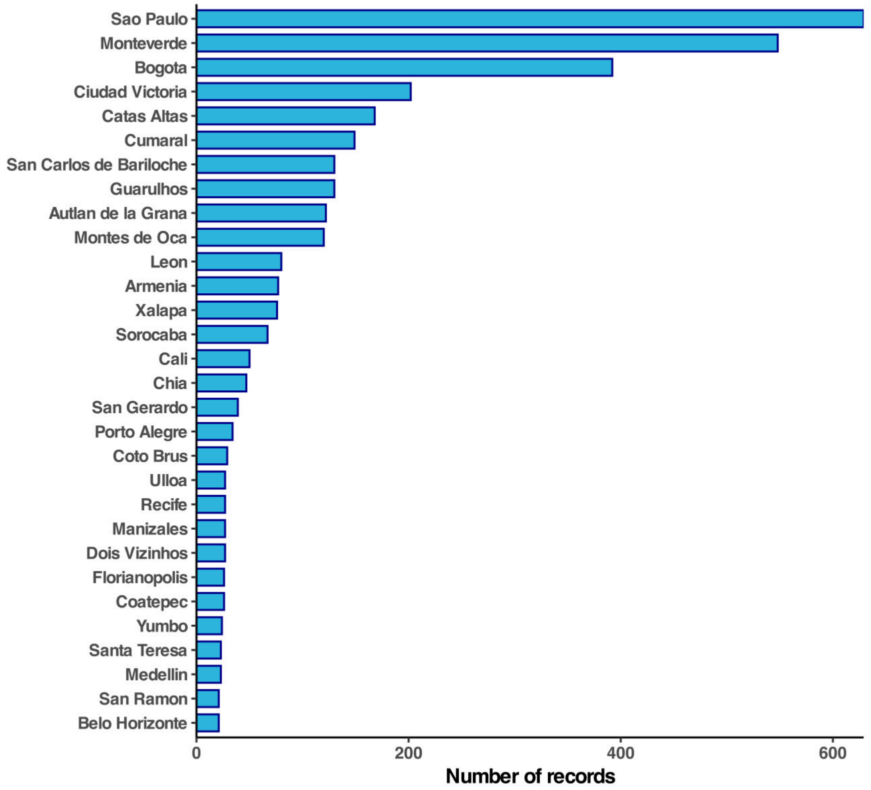

Figure 3. Municipalities with the highest number of bird–window collision records in the Neotropical region (1946–2020).

Although most post-collision data reports were dead specimens (2537 records), many individuals were alive when found (1515 records; Fig. 6a). Among the 1121 collided birds from sexually dimorphic species identified by sex, 648 were male and 473 were female (Fig. 6b). Regarding age, among the 2082 individuals with defined age, most affected by collisions with windows were adults (1682 records), while juveniles were fewer (400 records; Fig. 6c). A total of 2869 of the recorded collisions occurred on windows not provided of preventive measures (deterrents), while 389 occurred in panes equipped with some device aimed designed to mitigate bird–window collisions (Fig. 6d).

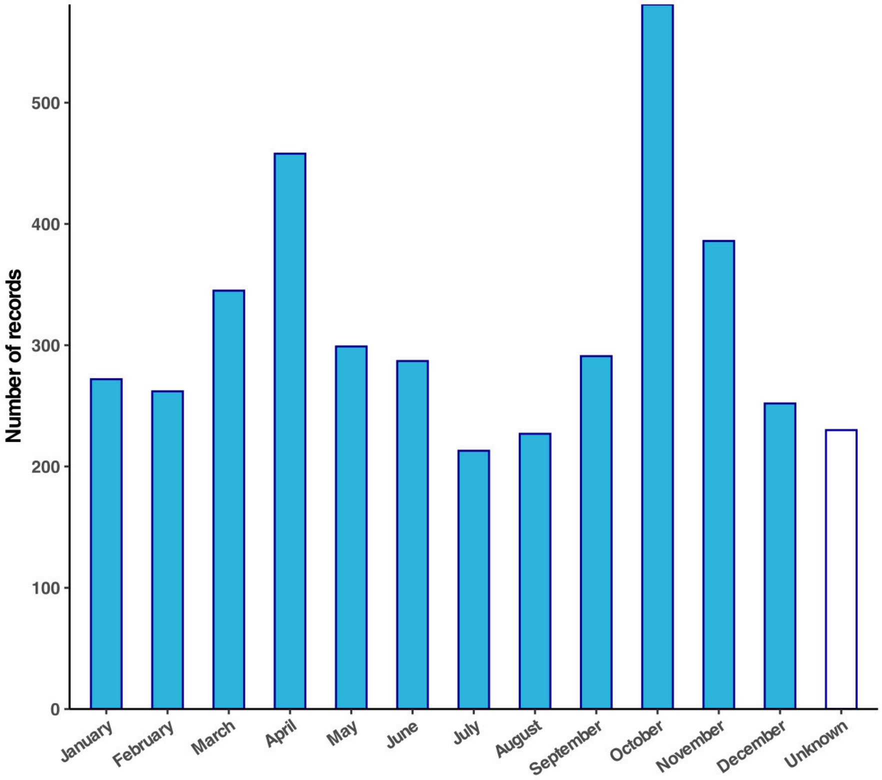

Figure 4. Monthly variation in bird–window collision records in the Neotropical region (1946–2020).

Most collisions (1919) occurred in low-rise buildings (<2 stories), while fewer (1237) took place in taller buildings (>2 stories; Fig. 6e). The collected records show a similar number of collisions occurring on translucent (1573) and reflective (1137) surfaces (Fig. 6f). Most records in our dataset correspond to species classified as Least Concern on the 2024 IUCN Red List (IUCN 2024) (4030 records); however, 14 of the species in the dataset are classified as Near Threatened (53 records) and six as Vulnerable (20 records; Fig. 6g). Finally,

regarding the data collection methods, most records were obtained through systematic procedures (1419 records), followed by those collected by chance (1252 records), and by government agencies (742 records). The remaining records came from other collection methods (632 records), with the fewest (58 records) being unreported by the collectors (Fig. 6h; see Class V for more details on how the systematic data were obtained).

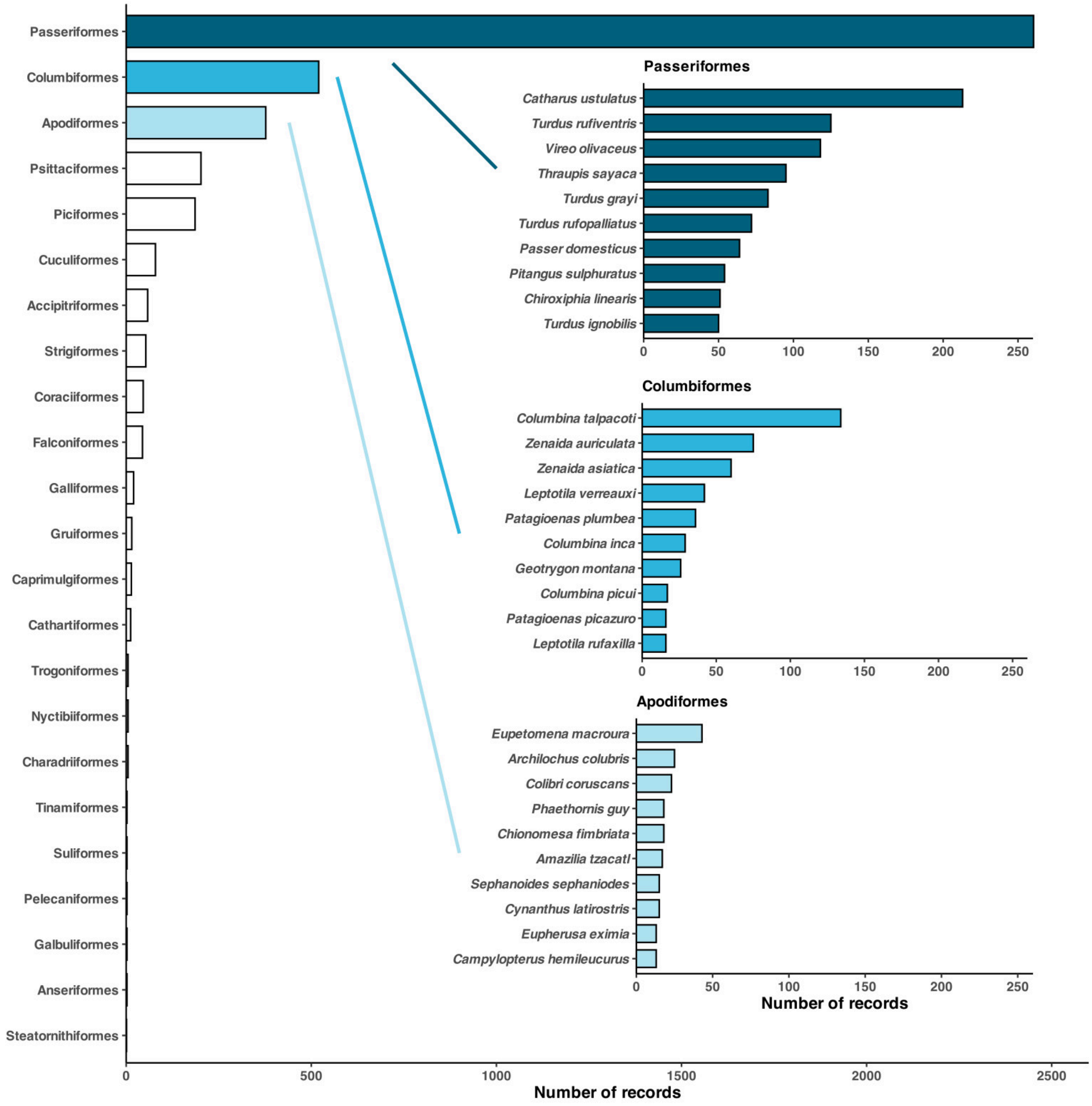

Figure 5. Bird–window collision records in the Neotropical region (1946–2020) by taxonomic order, with insets for top species in the orders with most records.

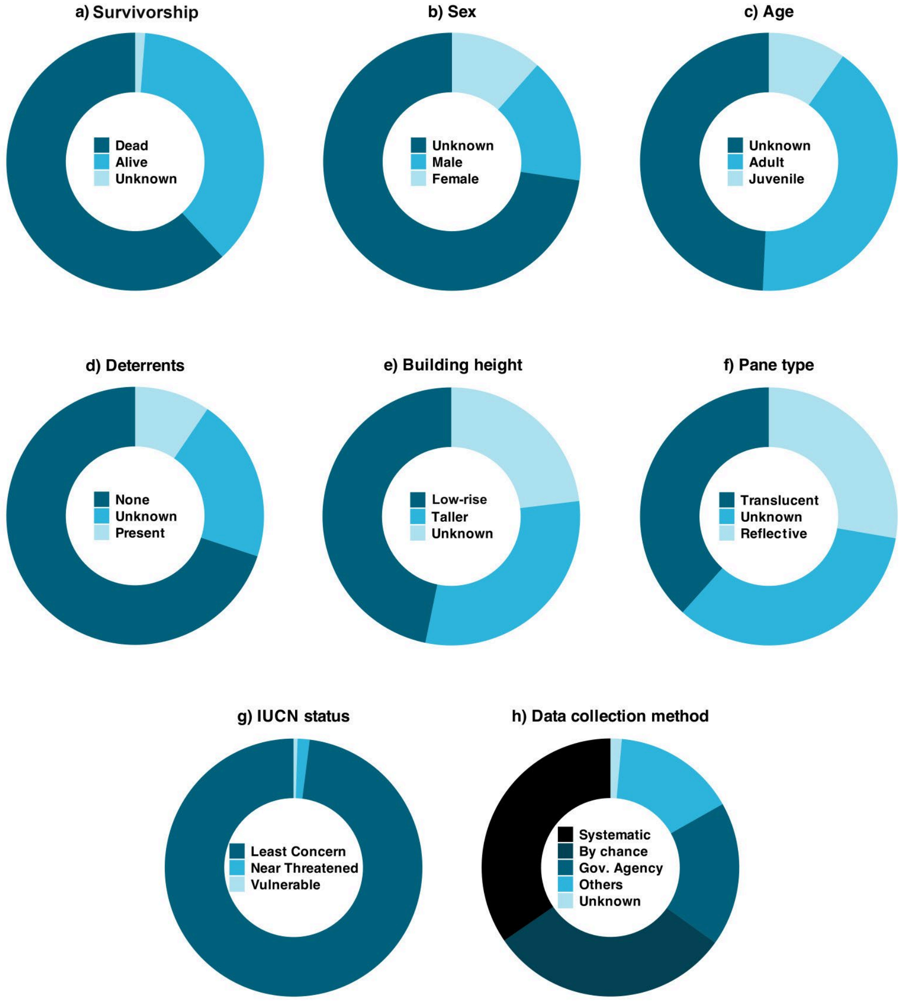

Figure 6. Bird–window collision records in the Neotropical region categorized by: a) survivorship, b) sex, c) age, d) presence of preventive deterrents, e) building height, f) pane type, g) IUCN status, and h) data collection method.

Class II. Research origin descriptors

A. ***Overall project description***

1. **Identity**: Bird–window collisions: A comprehensive dataset for the Neotropical region
2. **Originators:** Augusto João Piratelli, Bianca Costa Ribeiro, Ian MacGregor-Fors.
3. **Period of study**: 1946–2020.
4. **Objective**: Our primary objective was to develop a comprehensive and detailed dataset on bird–window collisions across the Neotropical region, incorporating both

published and unpublished sources. The database includes a taxonomic list along with specific details about each collision event, where information was available (e.g., data collection method, IUCN status, age, sex). Additional data, such as collision date, georeferenced location (country, state/province, locality), window type (translucent or reflective), building height (<2 or >2 stories), and the presence of preventive deterrents, were also included when possible.

5. **Sources of funding:** No financial aid was provided specifically for data compilation; however, several co-authors received support, as noted in the Acknowledgments.

B. ***Specific subproject description***

1. **Site description**
   a. *Neotropics*: This region spans approximately 20 million km² (~30°N to ~55°S; Udvardy, 1975) and includes all countries from Mexico in the north (while part of Mexico technically lies within the Nearctic region, the entire country was considered for this effort) to Chile and Argentina in the south (Morrone et al., 2022). The Neotropics, which encompass numerous biodiversity hotspots (*sensu* Myers et al., 2000), feature a remarkable diversity of ecosystems, ranging from sand dunes and tropical dry forests to pampas and Araucaria and coniferous forests (Olson et al., 2001; Morrone et al., 2022).
   b. *Countries*: Eleven Neotropical countries are represented in this database, listed in descending order by the number of records: Brazil, Colombia, Costa Rica, Mexico, Argentina, Ecuador, Venezuela, Chile, Uruguay, Panama, and Bolivia.
   c. *Main systems*: While urban areas are the primary ecosystems where bird–window collisions occur in the Neotropics and globally, the information in this dataset extends beyond cities to include buildings in smaller towns and even small developments typical of biological stations. This broader scope was enabled by an open call for data covering collision events throughout the biogeographic region.
2. **Data collection and curation**
   a. *Temporal coverage*: The dataset includes reports of bird–window strikes recorded from September 1946 to July 2020 (Fig. 1).
   b. *Data compilation*: To compile the most extensive dataset on bird–window collisions across the Neotropical region, we prepared a structured spreadsheet and invited potential contributors from all Neotropical countries via social media and email (see Class V). Contributions were welcomed regardless of whether collaborators were affiliated with educational institutions, research organizations, public agencies, or non-governmental organizations (NGOs). While we aimed for consistency by encouraging records to include species-level identification, location details (municipality, country, and geographic coordinates), and the date of occurrence (at least the year), a few exceptions

were made to maximize data collection. A few records missing some of these details were included to ensure the dataset captured the broadest possible range of collision events.

c. *Data curation*: We reviewed the database to ensure it contained as much information as possible while meeting key criteria, such as species-level identification. Our goal was to maximize the dataset by retaining records with the most complete information available. This review was conducted manually by at least two core team members.
d. *Location data*: We carefully verified geographic coordinates, cross-checking location details using Google Earth. Coordinates are reported with two decimal degrees (WGS84 reference system) to protect the privacy of those providing the data. The occurrence area for each reported species was validated by confirming its documented presence in the region using ornithological sources (e.g., Avibase, Wikiaves, eBird).
e. Taxonomy and common names: We standardized taxonomic nomenclature and common names in English following the guidelines of the American Ornithological Society for South American countries and territories, as well as that for North and Middle America (AOS, 2024a,b).

Class III. Dataset status and accessibility

A. ***Status***

1. **Latest update**: December 2020
2. **Latest archive date**: December 2020
3. **Metadata status**: Updated in December 2024
4. **Data verification**: Final data were verified by AJP and IM-F.

B. ***Accessibility***

1. **Storage location and medium**: The file 'BWC_Neotropical_region.csv', along with its associated metadata, is available for download with this data paper through the journal *Ecology* and in Zenodo at https://doi.org/10.5281/zenodo.15271126. The R script 'Glimpse to BWC Neotropical region dataset.R' for exploring the dataset can also be downloaded with this data paper through the journal *Ecology* and in the same Zenodo repository.
2. **Contact persons**: For general inquiries about the dataset, contact: Ian MacGregor-Fors (ian.macgregor@helsinki.fi).
3. **Copyright restrictions**: The 'BWC_Neotropical_region.csv' is free from copyright or proprietary restrictions. Please cite this data paper when using the data in publications or scientific presentations.

Class IV. Data structural descriptors

A. ***Dataset file***

1. **Identity**: BWC_Neotropical_region.csv.
2. **Format**: Comma-separated values (.csv).
3. **Size**: 894.1 kB (4103 rows excluding headers, 24 columns).
4. **Header information**: See Table 1.
5. **Data anomalies**: Information that could not be retrieved from contributors' shared data was classified as 'Unknown'. Notably, the dataset includes a relatively high number of missing cases for sex (73%) and age (49%), which is considered acceptable given that many species lack sexual dimorphism and age estimation was often challenging, particularly in reports from non-specialists. Moderate levels of missing information are present in three variables: pane type (34%), building height (23%), and the presence of preventive deterrents (21%). Finally, low-to-minimal missing data are found across other variables, including day (11%), month (6%), survivorship (1%), data collection method (1%), year (<1%), geographic location (latitude/longitude <1%), and locality (<1%).

B. ***Variable information***

1. Variable identity: See Table 1.
    a. Variable definition: See Table 1.

Table 1. Definitions and units of columns in the 'BWC_Neotropical_region.csv' dataset.

**Column name**	**Definition**	**Units / Levels**	**Example**
General.ID	Numeric identification of records	1–4103	311
Order	Taxonomic order based on the guidelines of the American Ornithological Society (AOS, 2024a,b)	Taxonomic order	Apodiformes
Family	Taxonomic family based on the guidelines of the American Ornithological Society (AOS, 2024a,b)	Taxonomic family	Trochilidae
Genus	Taxonomic genus based on the guidelines of the American Ornithological Society (AOS, 2024a,b)	Taxonomic genus	*Anthracothorax*
Species	Taxonomic species based on the guidelines of the American Ornithological Society (AOS, 2024a,b)	Scientific name	*Anthracothorax prevostii*
Common.name	Common English name based on the American Ornithological Society checklists (AOS, 2024a,b)	Common English name	Green-breasted Mango
IUCN.status	Conservation status reported of the species as reported in the International Union for Conservation of Nature (IUCN) Red List	Least Concern, Near Threatened, Vulnerable	Least Concern
Survivorship	The condition of the bird at the time the collision was encountered	Dead, Alive	Dead
Age	Visually assessed sexual maturity	Juvenile, Adult, Unknown	Adult
Sex	Sex visually determined based on sexual dimorphism	Female, Male, Unknown	Female
Year	Year the bird–window collision was recorded	1946–2020, Unknown	1981

Column name	Definition	Units / Levels	Example
Month	Month of the year the bird–window collision was recorded	January–December, Unknown	June
Day	Day of the month and year the bird–window collision was recorded	1–31, Unknown	12
Date.accuracy	Date accuracy of the collision is reported as: ‘exact’ (when the information was provided and confirmed), ‘approximate’ (when estimated by the data provider based on partial recollection), and ‘unknown’ (when no information was available)	Exact, Approximate, Unknown	Exact
Latitude	Geographic latitude of the reported collision, rounded to two decimals places to ensure privacy	UTM geographic coordinate system	19.30
Longitude	Geographic longitude of the reported collision, rounded to two decimals places to ensure privacy		-99.19
Location.accuracy	Besides rounding most data to two decimal places (which is why we refer to it as ‘precise’ rather than ‘exact’; see Latitude & Longitude above), there was also information that data providers masked in advance, which we categorize as ‘approximate’, and ‘unknown’ when no information was available	Precise, Approximate, Unknown	Precise
Locality	Locality (e.g., city, town, site) where the collision was reported to have occurred	Name of the locality	Mexico City
State.or.Province	State or Province, depending on the country’s nomenclature, where the collision was reported to have occurred	Name of the state or province	Sao Paulo
Country	Country where the collision was reported to have occurred	Name of the country	Colombia
Deterrent	Indicates whether or not a	Present, None,	Present

Column name	Definition	Units / Levels	Example
	preventive deterrent for bird–window collisions was present at the site of the collision	Unknown	
Building.height	Categorical description of the height of the building where the bird–window collision occurred: low-rise (<2 stories), taller (>2 stories).	Low-rise, Taller, Unknown	Low-rise
Pane.type	Type of window pane: reflective (mirrored or shiny surface) or translucent (allowing light to pass through, but not clear).	Reflective, Translucent, Unknown	Reflective
Data.collection.method	Method by which bird–window collision data were acquired: 'systematically' (following a specific local protocol; see Class V for details), 'by chance,' 'collected by Government Agencies' (e.g., zoos), or following a different approach as identified by data providers ('others,' see Class V for details).	Systematic, By Chance, Gov. Agency, Others, Unknown	Systematic

Class V. Supplemental descriptors

A. Data or acquisition methods

Information requested about the method used to collect the data:

How did you/your group collect the data you sent us?

( ) By chance / Anecdotally

( ) Systematic collection procedure (e.g., research/survey)

( ) Received by government agency (e.g., zoos)

( ) Others

If your answer was ‘Systematic collection procedure (e.g., research/survey),’ please briefly explain how.

If you answer was ‘others,’ please briefly explain.

Result

We received a response indicating that 98.5% of the bird–window collision records were collected. Below is a summary of the systematic procedure used to compile the 1419 records in our dataset, which were provided by the responding colleagues.

- Monthly visits were conducted to different locations within [city], with trips lasting from 1 to 4 hours. The purpose was to observe and document any dead birds found on the ground. The number of observers ranged from 6 to 15.
- The data collection was somewhat systematic. Security guards were asked to collect carcasses, and some buildings were surveyed almost daily (excluding weekends). However, the approach was not fully anecdotal but also not fully systematic.
- At the [local bird observatory], cases of bird–window collisions were recorded. Special bird stamps were used on windows to prevent collisions, but all cases were still recorded.
- An active search was conducted among 29 buildings of the researched institution. The route was randomly defined, covering twice a day, and data were recorded on a spreadsheet. Specifically, buildings with large areas of windows or glass were selected, toured after 3 pm in both directions, and bird carcasses within 2 meters from the base of the building were recorded.
- In [city] a study was conducted over several years, with biweekly sampling at three sites. The search and collection of biological material were done three times a month, with established routes around the buildings. Carcasses were sought at 20 meters from the base of the buildings between 7:00 am and 12:00 pm.
- At the [local botanical garden] inspections were conducted three times a week between 2:00 pm and 4:00 pm from August to November 2015 and from February to May 2016.

The buildings and their surroundings within a 2-meter radius were searched for bird carcasses. Visitors, officials, and operators were encouraged to report collisions.

- Following Hager et al., (2017), a stratified sampling approach was used for most individuals. Six buildings of varying sizes were identified. Carcass surveys were conducted twice a week between 10:00 am and 4:00 pm. Observers made two complete passes around each building, one clockwise and one counterclockwise. Any bird carcasses found were immediately collected and identified. Additionally, chance encounters reported by professors, students, or others were included in the data. Carcasses were taken to the [local University] for bird identification.
- Transect surveys were conducted around the university campus.
- Standardized revision of focal buildings was performed.
- In [city], monthly collections were conducted twice a month for one year in urban areas.
- Tours around the building were conducted twice a day, in the morning at 9 am and in the afternoon at approximately 2 pm. The data collection period lasted for six months.
- Daily surveys involved observers making two complete passes, one clockwise and one counterclockwise, around the perimeter of each building. Each survey was conducted by one or two observers. In the case of a single observer, they walked the perimeter in one direction and then made a second pass in the opposite direction. Any bird carcasses found during single-observer surveys were documented and collected immediately. For further details, refer to Hager et al. (2017).
- Daily surveys were conducted during two periods: from April 2016 to March 2017, and from June 24 to July 5, 2019. Surveys were carried out in the morning from 10:00 am to 11:30 am and in the afternoon from 6:30 pm to 8:00 pm.
- Following the standardized protocol proposed by Hager and Cosentino (2014), daily surveys were conducted around focal buildings to search for and collect all bird carcasses within 2 meters from the edge of each building facade.
- Daily surveys were conducted around focal buildings with the aim of searching for and collecting all bird carcasses within 2 meters from the edge of each building facade.
- Data collection for bird–window collisions was based on the institution's database. specifically for birds with a history of rescue after collisions. Cases with compatible symptomatology but no collision history were not included.
- The individual stating the information is the curator of the zoology department at the [local science museum]. Some specimens in their collection forms are related to bird–window collisions and their context of death.

Below is the information provided by the colleagues who responded ‘others,’ corresponding to 15% of the records in our dataset.

- In [location], the community members were asked to collect and bring bird carcasses that collided with their buildings to designated fridges. The collected bodies were then identified and information such as age, sex, location of the building, date, and the person who found and collected the bird was recorded.
- An individual frequently observes a glass wall while riding a bicycle and looks for dead birds there.
- Bird species were recorded randomly whenever they collided with the glass panes, but there was no systematic approach for the research.
- Dead birds were systematically collected to assess the impact of new buildings at the [local university]. Any data older than 2018 were coincidental as they were brought to the zoology museum of the biology department.
- The buildings at the individual's institution are covered by windows, and reported collisions resulting in bird deaths are registered.
- The data submitted includes occasional records and information from an online survey conducted during the mandatory COVID-19 lockdown in [country] in 2020. People surveyed through various media platforms were asked to report bird collisions with windows in their homes during a 15-day period.
- In 2018/2019, the individual built a house with a 7-meter glass wall and large glass windows. Over time, they noticed bird collisions and deaths on the glass. Initially, the records were random and based on images only. Additionally, data sent to this survey includes those from walking around [city], they would spot birds (as the collision points were along their daily route) and record data such as the location (address, type of structure, attractions, and bird condition).
- Cases of bird collisions were documented based on animals received by the [local wildlife division]. The service started in 1991, but from 2016 onwards, the cause of trauma, including collisions with glass, was better documented to ensure such information was not overlooked.

Acknowledgments
We thank the anonymous reviewers and editor Kathryn L. Cottingham for their valuable suggestions, which significantly improved the quality of the manuscript. We are also deeply thankful to the Federal University of São Carlos, Botanic Garden of Bogotá "José Celestino Mutis", Center for Biodiversity and Biotechnology Studies of Quindío, Instituto Espaço Silvestre, Secretary of Environment and Sustainability of Sorocaba (SP, Brazil), Museum of Natural Sciences of the Secretary of the Environment of Rio Grande do Sul - Brazil, Private Reserve of the Natural Heritage Sanctuary of Caraça in Minas Gerais Brazil, Museum of Zoology of the University of Costa Rica, Environment and Climate Change Canada and Klamath Bird Observatory, Western Hummingbird Partnership and Urban Bird Program of the National Commission for the knowledge and use of biodiversity (PAU-CONABIO) of Mexico for providing resources and/or support to scientific research. We also thank our colleagues Liguori L., Herazo B., Perrier D., Perrier V., Segura J.E.S., Klem Jr. D., Arévalo E., Ulate R., Cervantes L.E.R., Rosselli L., Córdoba S., Olvera Acosta RB., Crispim L., Mello M.G., Lopes A., Silva D., Yamaguchi A. and Tortato and Ocampo-Peñuela families for help at data collection and/or providing their databases. We also appreciate the academic communities such as students, professors, and secretaries of the Federal University of São Carlos, Faculty of Environmental and Rural Studies, Javeriano Museum of Natural History, to the Institute National University of Sciences (ICN) and, to the PUJ Cleaning and Surveillance staff for their important collaboration. To the students of Zoology 2 at the University of Costa Rica and the students of Engineering in Natural and Agricultural Resources for their collaboration in the data collection. A. J. Piratelli thanks the National Council for Scientific and Technological Development (CNPq) for financial support (#316032/2023-9).

REFERENCES

(AOS) American Ornithological Society. 2025. Species Lists of Birds for South American Countries and Territories. Chicago: American Ornithological Society. https://americanornithology.org/publications/north-and-middle-american-checklist/

ajpiratelli. 2025. ajpiratelli/Data-Paper-BWC: Dataset and code for: "BIRD-WINDOW COLLISIONS: a comprehensive dataset for the Neotropical region" (doi-release). Zenodo. https://doi.org/10.5281/zenodo.15271127

Agudelo-Álvarez, L., J. Moreno-Velasquez, and N. Ocampo-Peñuela. 2010. "Colisiones de aves contra ventanales en un campus universitario de Bogotá, Colombia". Ornitología Colombiana 10: 3–10.

Al-Kodmany, K. 2013. "The Visual Integration of Tall Buildings: New Technologies and the City Skyline". Journal of Urban Technology 20: 25–44. doi.org/10.1080/10630732.2012.735481.

Basilio, L. G., D. J. Moreno, and A. J. Piratelli. 2020. "Main causes of bird-window collisions: a review. Anais da Academia Brasileira de Ciências". 92: e20180745. doi.org/10.1590/0001-3765202020180745

Gaston, K. J., T. M. Blackburn, and K. K. Goldewijk. 2003. "Habitat conversion and global avian biodiversity loss. Proceedings of the Royal Society of London". Series B: Biological Sciences 270: 1293–1300. doi.org/10.1098/rspb.2002.2303

Gómez-Martínez, M. A., D. Klem, O. Rojas-Soto, F. González-García, and I. MacGregor-Fors. 2019. "Window strikes: bird collisions in a Neotropical green city". Urban Ecosystems 22: 699–708. doi.org/10.1007/s11252-019-00858-6

Hager, S., B. J. Cosentino, M. A. Aguilar-Gómez, M. L. Anderson, M. Bakermans, T. J. Boves, et al. 2017. "Continent-wide analysis of how urbanization affects bird-window collision mortality in North America". Biological Conservation 212: 209–2015. doi.org/10.1016/j.biocon.2017.06.014

Hager, S., and B. J. Cosentino. 2014. "Surveying for bird carcasses resulting from window collisions: a standardized protocol". Peer-J Preprints 2: e406v1. doi.org/10.7287/peerj.preprints.406v1

Jiménez-Peñuela, J., M. Ferraguti, J. Martínez-de La Puente, R. C. Soriguer, and J. Figuerola. 2021. "Urbanization effects on temporal variations of avian haemosporidian infections". Environmental Research 199: 111234. https://doi.org/10.1016/j.envres.2021.111234

Klem, D. 2006. "Glass: A Deadly Conservation Issue for Birds". Bird Observer 34: 73–81.

Klem, D. 2015. "Bird–Window collisions: A Critical Animal Welfare and Conservation Issue". Journal of Applied Animal Welfare Science 18: S11–S17. doi.org/10.1080/10888705.2015.1075832

La Sorte, F. A., C. A. Lepczyk, M. F. J. Aronson, M. A. Goddard, M. Hedblom, M. Katti, I. MacGregor-Fors, U. Mörtberg, C. H. Nilon, P. S. Warren, N. S. G. Williams, and J. Yang. 2018. "The phylogenetic and functional diversity of regional breeding bird assemblages is

reduced and constricted through urbanization". Diversity and Distributions 24: 928–938. doi.org/10.1111/ddi.12738

Lee, S. J., F. N. Matos, C. R. R. Gonzaga, M. A. de Medeiros, S. de F. S. Leandro, R. H. F. Teixeira, A. L. M. da Costa, and A. J. Piratelli. 2024. "Post mortem analysis of birds that collided with glass panes reveals multiple injuries and fractures". Ornithology Research 32: 399–403. doi.org/10.1007/s43388-024-00201-4

Loss, S. R., T. Will, S. S. Loss, and P. P. Marra. 2014. "Bird–building collisions in the United States: Estimates of annual mortality and species vulnerability". The Condor 116: 8–23. https://doi.org/10.1650/CONDOR-13-090.1

MacGregor-Fors, I., and J. F. Escobar-Ibáñez. 2017. Birds from Urban Latin America, Where Economic Inequality and Urbanization Meet Biodiversity. Pages 1–10 in I. MacGregor-Fors and J. F. Escobar-Ibáñez, editors. Avian Ecology in Latin American Cityscapes. Springer International Publishing, Cham. doi.org/10.1007/978-3-319-63475-3_1

MacGregor-Fors, I., M. García-Arroyo, and J. Quesada. 2022. "Keys to the city: an integrative conceptual framework on avian urban filtering". Journal of Urban Ecology juac026: 1-5 doi.org/10.1093/jue/juac026

Maxwell, S. L., R. A. Fuller, T. M. Brooks, and J. E. M. Watson. 2016. "Biodiversity: The ravages of guns, nets and bulldozers". Nature 536: 143–145. doi.org/10.1038/536143a

Mitrus, C., and A. Zbyryt. 2018. "Reducing avian mortality from noise barrier collisions along an urban roadway". Urban Ecosystems 21: 351–356. doi.org/10.1007/s11252-017-0717-7

Morrone, J. J., T. Escalante, G. Rodríguez-Tapia, A. Carmona, M. Arana, and J. D. Mercado-Gómez. 2022. "Biogeographic regionalization of the Neotropical region: New map and shapefile". Anais da Academia Brasileira de Ciências 94: e20211167. doi.org/10.1590/0001-3765202220211167

Myers, N., R. A. Mittermeier, C. G. Mittermeier, G. A. B. Da Fonseca, and J. Kent. 2000. "Biodiversity hotspots for conservation priorities". Nature 403: 853–858. https://doi.org/10.1038/35002501

Olson, D. M., E. Dinerstein, E. D. Wikramanayake, N. D. Burgess, G. V. N. Powell, E. C. Underwood, J. A. D'amico, I. Itoua, H. E. Strand, J. C. Morrison, C. J. Loucks, T. F. Allnutt, T. H. Ricketts, Y. Kura, J. F. Lamoreux, W. W. Wettengel, P. Hedao, and K. R. Kassem. 2001. "Terrestrial Ecoregions of the World: A New Map of Life on Earth". BioScience 51: 933. doi.org/10.1641/0006-3568(2001)051[0933:TEOTWA]2.0.CO;2

Palacio, F. X., L. M. Ibañez, R. E. Maragliano, and D. Montalti. 2018. "Urbanization as a driver of taxonomic, functional, and phylogenetic diversity losses in bird communities". Canadian Journal of Zoology 96: 1114–1121. doi.org/10.1139/cjz-2018-0008

Pearman, M., J. Freile, J. Miranda, and V. Remsen. 2025. Species Lists of Birds for South American Countries and Territories. Baton Rouge: International Ornithologists' Union. https://www.museum.lsu.edu/~Remsen/SACCCountryLists.htm

Rebolo-Ifrán, N., L. Zamora-Nasca, and S. A. Lambertucci. 2021. "Cat and dog predation on birds: The importance of indirect predation after bird-window collisions". Perspectives in Ecology and Conservation 19: 293–299. doi.org/10.1016/j.pecon.2021.05.003

Ribeiro, B. C., and A. J. Piratelli. 2020. "Circular-shaped decals prevent bird-window collisions". Ornithology Research 28: 69–73. doi.org/10.1007/s43388-020-00007-0

Rössler, M., E. Nemeth, and A. Bruckner. 2015. "Glass pane markings to prevent bird-window collisions: less can be more". Biologia 70: 535–541. doi.org/10.1515/biolog-2015-0057

Sabo, A. M., N. D. G. Hagemeyer, A. S. Lahey, and E. L. Walters. 2016. "Local avian density influences risk of mortality from window strikes". PeerJ 4: e2170. doi.org/10.7717/peerj.2170

Santiago-Alarcon, D., and C. A. Delgado-V. 2017. Warning! Urban Threats for Birds in Latin America. Pages 125–142 in I. MacGregor-Fors and J. F. Escobar-Ibáñez, editors. Avian Ecology in Latin American Cityscapes. Springer International Publishing, Cham. doi.org/10.1007/978-3-319-63475-3_7

Sol, D., C. González-Lagos, D. Moreira, J. Maspons, and O. Lapiedra. 2014. "Urbanisation tolerance and the loss of avian diversity". Ecology Letters 17: 942–950. doi.org/10.1111/ele.12297

Udvardy, M. D. F. 1975. A classification of the biogeographical provinces of the world. IUCN Occasionaal Paper No. 18. Gland: International Union for Conservation of Nature. https://iucn.org/sites/default/files/import/downloads/udvardy.pdf

Uribe-Morfín, P., M. A. Gómez-Martínez, L. Moreles-Abonce, A. Olvera-Arteaga, H. Shimada-Beltrán, and I. MacGregor-Fors. 2021. "The invisible enemy: Understanding bird-window strikes through citizen science in a focal city". Ecological Research 36: 430–439. doi.org/10.1111/1440-1703.12210

Żmihorski, M., D. Kotowska, and E. Zyśk-Gorczyńska. 2022. "Using citizen science to identify environmental correlates of bird-window collisions in Poland". Science of The Total Environment 811: 152358. doi.org/10.1016/j.scitotenv.2021.152358
